# Edge State Quantum Interference in Twisted Graphitic Interfaces

**DOI:** 10.1002/advs.202102261

**Published:** 2022-03-13

**Authors:** Annabelle Oz, Debopriya Dutta, Abraham Nitzan, Oded Hod, Elad Koren

**Affiliations:** ^1^ Department of Physical Chemistry School of Chemistry The Raymond and Beverly Sackler Faculty of Exact Sciences and The Sackler Center for Computational Molecular and Materials Science Tel Aviv University Tel Aviv 6997801 Israel; ^2^ Faculty of Materials Science and Engineering and the Russell Berrie Nanotechnology Institute Technion – Israel Institute of Technology Haifa 3200003 Israel; ^3^ Department of Chemistry University of Pennsylvania Philadelphia PA 19103 USA; ^4^ The Nancy and Stephen Grand Technion Energy Program Technion – Israel Institute of Technology Haifa 3200003 Israel

**Keywords:** 2D materials, edge states, graphene interfaces, quantum interference, transport

## Abstract

Zigzag edges in graphitic systems exhibit localized electronic states that drastically affect their properties. Here, room‐temperature charge transport experiments across a single graphitic interface are reported, in which the interlayer current is confined to the contact edges. It is shown that the current exhibits pronounced oscillations of up to ≈40 µA with a dominant period of ≈5 Å with respect to lateral displacement that do not directly correspond to typical graphene lattice spacing. The origin of these features is computationally rationalized as quantum mechanical interference of localized edge states showing significant amplitude and interlayer coupling variations as a function of the interface stacking configuration. Such interference effects may therefore dominate the transport properties of low‐dimensional graphitic interfaces.

## Graphical abstract

Graphitic systems with zigzag edges exhibit strongly confined electronic edge states. Here, room‐temperature charge transport experiments across a laterally confined twisted graphitic interface are reported. It is shown that the current exhibits oscillations with a dominant period of ≈5 Å that do not directly correspond to graphene lattice spacing, but originates from quantum mechanical interference between localized edge states.

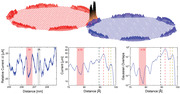

## 1 Introduction

Graphene, probably the most studied material of the last decade, manifests extraordinary electronic and mechanical characteristics. Properties such as high charge carrier mobility,^[^ ^1^, ^2^ ^]^ the possibility of controlling the energy gap by applying vertical electric fields,^[^ ^3^, ^4^, ^5^ ^]^ and the rich physics involved in its various stacking configurations^[^ ^6^, ^7^, ^8^ ^]^ such as superlubricity^[^ ^9^, ^10^, ^11^, ^12^, ^13^, ^14^ ^]^ and the appearance of superconductivity in magic‐angle twisted bilayers^[^ ^7^, ^15^ ^]^ make graphitic interfaces promising candidates for future applications in nanotribology, nanoelectromechanics, and nanoelectronics. Graphitic systems with bare (or chemically passivated) zigzag edges exhibit strongly confined electronic edge states with a corresponding sharp zero‐energy peak in their density of states (DOS).^[^ ^16^, ^17^, ^18^, ^19^, ^20^, ^21^ ^]^ Due to the unique nature of their wave function, these edge states manifest exotic properties, such as topologically protected subgap conductivity in bilayer graphene,^[^ ^19^, ^22^ ^]^ electric‐field tunable magnetism,^[^ ^17^, ^23^ ^]^ and valley‐dependent transport.^[^ ^24^, ^25^ ^]^ Such edges, however, may also pose challenges for practical applications, including edge leakage that is expected to limit the functionality of graphene‐based logic devices,^[^ ^22^, ^26^, ^27^ ^]^ and topologically protected metallic edge states that decrease the effectiveness of topological insulators.^[^ ^19^, ^28^ ^]^ To overcome these challenges, a better understanding of the interplay between edge and bulk transport properties and of edge‐based interlayer transport in confined bilayer interfaces is required.

Recently, we presented an experimental setup based on atomic force microscopy (AFM), which enables precise control over the stacking configuration of a single twisted graphitic interface.^[^ ^10^ ^]^ This allowed us to study the intricate interplay between the misfit angle of the interface and its transport properties^[^ ^6^ ^]^ and to quantitatively distinguish between bulk and edge interlayer transport contributions.^[^ ^22^ ^]^ In the present work, we utilize this unique setup to study the interlayer charge transport properties of a single twisted graphitic interface, in which the current is confined to the junction's edges. We observe pronounced current fluctuations as a function of the lateral shear configuration of the interface with a dominant period of ≈5 Å for systems of confined interlayer overlap of magnitude ≤ 5 nm. Notably, these fluctuations decay rapidly with increasing interlayer overlap. Using transport calculations based on the Landauer formalism and a tight‐binding Hamiltonian, we show that the observed current fluctuations correspond to quantum mechanical interference, manifested by substantial fluctuations of the wave function probability amplitudes. As a result, the interlayer edge transport does not directly correspond to the interlayer lattice registry and exhibits a surprisingly richer behavior.

## 2 Results

### 2.1 Experimental Analysis

**Figure 1:**
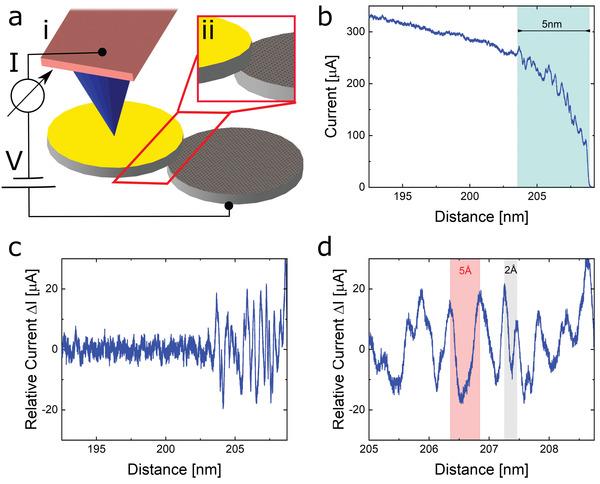
Electromechanical manipulation of a nano‐sized graphitic mesa structure at the edge contact regime. a) Schematic illustration of the electromechanical manipulation using an AFM apparatus (i) enabling to achieve high lateral precision near the interface edges (ii). b) Total measured current as a function of lateral displacement near the mesa edges. Strong current fluctuations are observed along the last 5 nm and are associated with pronounced quantum mechanical interference effects of the edge states. A constant voltage of 2.5 V was applied to the AFM tip and the HOPG substrate was grounded. c,d) Relative current, presented with respect to the average background current profile, Δ*I*  =  *I* − *I*_Average_, displayed as a function of lateral displacement measured along the last 15 and 5 nm from the mesa edge, respectively. The average background current of a given point along the measured profile was calculated using a window of 50 points around it. Pronounced current fluctuations with an amplitude of up to ≈40 µA arise with dominant spatial periodicity of ≈5 Å along with fewer fluctuations showing ≈2 Å peak separation (d).

Current confinement to the interface edges was achieved by electromechanical manipulation of nano‐sized contacts using atomic force microscopy (AFM) in conjugation with charge transport measurements (see **Figure** **1**a). Graphitic contacts featuring cylindrical structures with a typical height of 50 nm and a diameter of 200 nm were constructed from highly oriented pyrolytic graphite (HOPG) based on a recently presented fabrication method.^[^ ^6^, ^10^, ^29^, ^30^, ^31^ ^]^ Pd and Au metal layers of 10 and 40 nm, respectively, were used as self‐aligned shadow masks and top metallic contacts. Nanomanipulation of individual nano‐sized graphitic contacts was performed under ambient conditions and the electrical connection to the top metallic contact was made via a Pt/Ir metal‐coated AFM tip that was cold‐welded by applying a normal force of 50 nN along with an electrical current pulse of ≈1 mA for a duration of 1 s. The strong mechanical contact formed allows to apply lateral shear forces inducing a shear glide along a single basal plane within the graphitic stack.^[^ ^10^ ^]^ The lateral shear force was continuously monitored to ensure that the sliding was performed under superlubric conditions,^[^ ^10^, ^12^, ^13^, ^32^ ^]^ indicating that the sliding graphitic interface was twisted by a rotational mismatch angle of ≈ 10° ± 5° (see Section S1, Supporting Information).^[^ ^10^, ^31^ ^]^ During the mechanical manipulation, a DC bias voltage was applied to the AFM tip and the vertical current passing through the entire structure was measured using a pre‐amplifier that collected the current from the HOPG substrate.

Figure 1b presents the measured total current as a function of lateral displacement along the last ≈15 nm of the slide before full removal of the top stack from the bottom mesa. The entire current profile starting from the fully eclipsed configuration between the top and the bottom mesa stacks up to full removal is presented in Figure S1 (Section S2, Supporting Information). Pronounced current fluctuations with a magnitude of up to ≈ 40 µA and a dominant period of ≈5 Å are observed throughout the final 5 nm displacement before reaching the mesa edge (Figure 1c,d). A few weaker current fluctuations with a shorter period of ≈ 2 Å are also observed. The magnitude of the interface conductance variation along a single current fluctuation was extracted using a numerical fit to the measured current, based on the corresponding equivalent electrical circuit (see Section S2, Supporting Information) yielding a value of Δ*G*  =  2 ± 0.5  × 10^−5^ [*S*]  ≈  0.25  × *G*_0_. Interestingly, the onset of the current fluctuations was observed below an interface conductance value of ≈2  × *G*_0_.

The interfaces considered herein consist of single crystalline planar graphitic layers, as is evident by their observed superlubric characteristics.^[^ ^6^, ^10^, ^22^, ^31^ ^]^ Hence, the loss of interference signal beyond an overlap range of a few nanometers cannot be rationalized by the room temperature in‐plane electronic mean free path, which can reach hundreds of nanometers in high quality graphitic structures.^[^ ^33^, ^34^ ^]^ Notably, a similar range of interference effects in graphitic interfaces was recently reported in a break‐junction study manipulating bilayer graphene at room temperature, where the measured current periodicity of ≈6.9 Å was interpreted as a beating pattern of two lower periodicities (2.46 and 3.69 Å) that were theoretically predicted for an AB stacked graphene bilayer.^[^ ^35^ ^]^ These lower periodicities were associated with commensuration and Fabry–Pérot‐like interference effects of the electronic wave functions, respectively. This interpretation of the mismatch between the period of the observed current fluctuations and the lattice parameter of graphene (*a*  =  2.46 Å) indicates that simple geometric considerations, which can rationalize unique bulk transport characteristics,^[^ ^6^, ^22^ ^]^ are insufficient to understand the observed edge transport behavior. Therefore, careful attention should be given to the interlayer electronic coupling in edge‐based transport through such atomic constrictions, and to interference effects between different electronic pathways. In this respect, several scanning tunneling microscopy experiments,^[^ ^20^, ^21^ ^]^ supported by elaborate calculations,^[^ ^19^ ^]^ have demonstrated substantial increase in the local density of states (LDOS) over a range of 2–5 nm near zigzag graphitic edges. This provides strong indication of the crucial role of zigzag edge states in the interlayer transport characteristics of edge‐overlapping graphitic surfaces.

### 2.2 Theoretical Analysis

To unveil the origin of the observed current oscillations in our twisted graphene interface, their lower periodicity (≈5 Å) with respect to that previously observed for bilayer graphene,^[^ ^35^ ^]^ and the relation to interlayer electronic coupling between localized edge states, we examined their transport properties, as well as the variations of their electronic wave function, upon shifting between different stacking configurations. To this end, we constructed 10° twisted (with respect to the Bernal stacked configuration) circular bilayer graphene models, in agreement with the expected experimental interlayer twist angle,^[^ ^10^, ^22^ ^]^ and described their electronic properties using a tight‐binding Hamiltonian that includes an exponentially decaying inter‐layer hopping integral (see Section S3, Supporting Information).^[^ ^6^, ^36^ ^]^ For each stacking configuration we calculated the full interlayer electronic transmittance probability using the non‐equilibrium Green's function formalism (see Section 4, Supporting Information).^[^ ^37^ ^]^ This allowed us to evaluate the full current versus interlayer displacement profile via the Landauer formalism.^[37]^ Furthermore, to obtain spatially resolved information we calculated the molecular orbitals (MOs) by diagonalizing the corresponding Hamiltonian. For each eigenstate residing within the Fermi transport window (set by the external bias potential) we multiplied its absolute squared MO expansion coefficients by the transmittance probability evaluated at the corresponding eigenvalue energy. This effectively weighs each MO according to its contribution to the transport process. To visualize the interlayer overlap between the transmittance‐weighted MOs, we assigned each atomic site a two‐dimensional Gaussian function of a fixed height, whose width is set proportional to the sum of transmittance‐weighted coefficients of all MOs within the bias window associated with this atomic position (see Section 3, Supporting Information for further details).

**Figure 2:**
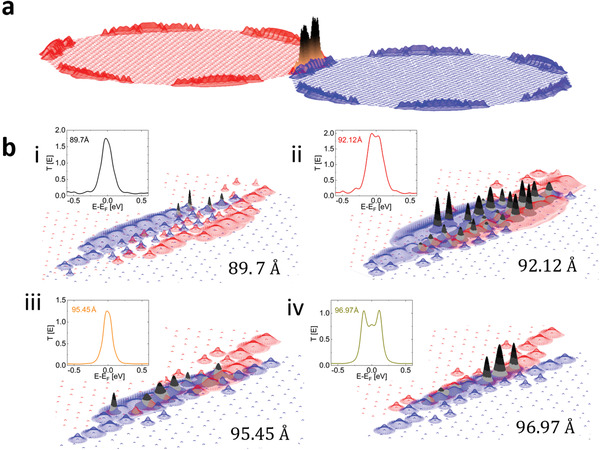
a) Map of the two‐dimensional Gaussian functions representing the sum of transmittance‐weighted MO coefficient at each atomic site of the upper (red) and lower (blue) 10 nm diameter circular graphene flakes in a 10° twisted bilayer. The Gaussians shown in panel (a) are scaled by ×10 for better visualization. b) Zoom‐in on the overlap region of the system shown in (a) for four lateral displacements of: 89.7 and 95.45 Å, where the calculated current presents local minima (30.1 and 19.1 µA, respectively), and 92.12 and 96.97 Å, where the calculated current presents local maxima (47.8 and 26.4 µA, respectively). See also Figure 3 below. The corresponding energy‐dependent transmittance probabilities are plotted in the insets.

**Figure** **2**a depicts such a calculation for a 10° twisted bilayer system composed of two 10 nm diameter circular graphene flakes shifted by 92.12 Å. The two‐dimensional Gaussian functions associated with atomic sites of the top (red) and bottom (blue) flakes illustrate the contribution of each atomic position to the interlayer transport of the interface, clearly manifesting the enhanced contribution of the zigzag edges. The overlaps between the Gaussian functions associated with atomic sites of the lower and upper flakes are shown in black (fading to orange for clarity of the representation), demonstrating the spatial region governing the vertical electronic transport through the bilayer. Figure 2b presents a zoom‐in on the contact area of the same bilayer flake for four lateral shifts of 89.7, 92.12, 95.45, and 96.97 Å. Edge state contributions are clearly manifested for all four configurations; however, the degree of interlayer overlap between the atomic Gaussians associated with the upper and lower flake edge atoms shows significant variations with the lateral position. Specifically, pronounced overlaps are obtained for lateral shifts of 92.12 and 96.97 Å, whereas smaller overlaps are observed for the 89.7 and 95.45 Å positions. The degree of overlap correlates with the transmittance probability plots (shown as insets at each position), which integrate to yield higher current values for the lateral positions in which the overlaps are larger. Movie S1 (Supporting Information) presents the wave function amplitude variations of an individual edge state next to the Fermi Energy as a function of interlayer position, demonstrating significant fluctuations of the wave function amplitudes with interlayer shifts.

**Figure 3:**
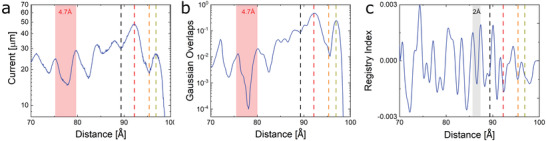
Computational investigation of interlayer current and registry variations in a twisted graphitic interface at the edge contact regime. a) Vertical current as a function of lateral shift, calculated at a bias voltage of 0.8 V for a 10 nm diameter circular bilayer graphene system twisted by 10° from Bernal stacking with a fixed interlayer distance of 3.35 Å. b) Lateral position dependence of the summed overlaps between the two‐dimensional Gaussians representing the atomic‐centered transmittance‐weighted MO coefficients of the upper and lower flakes within the Fermi transport window. c) Registry index variations with lateral shift. The moderately varying RI background was subtracted in order to clearly present the RI fluctuations (see Section S5, Supporting Information, for further details). A lateral distance of 0 Å indicates that the flakes are completely overlapping, whereas at a lateral distance of 100 Å the upper flake is fully removed from its lower counterpart. The four lateral shifts of 89.7, 92.12, 95.45, and 96.97 Å, studied in Figure 2, are marked here in dashed black, red, orange, and green lines, respectively.

The dependence of the transmittance‐weighted atomic‐centered Gaussian overlaps on the lateral shift manifests an intricate interplay between geometrical interlayer registry variations and quantum mechanical electronic coupling modulations. The combined effect of both contributions dictates the vertical electronic transport characteristics of the laterally shifted bilayer system. To demonstrate this, we use the transmittance probability curves, calculated to construct the transmittance‐weighted Gaussians at various shift positions, to evaluate the Landauer vertical current through the bilayer flake (more details regarding the calculations are provided in Section S4, Supporting Information).^[^ ^6^, ^22^, ^36^ ^]^ **Figure** **3**a presents the interlayer current versus the relative lateral interlayer displacement of the 10° twisted bilayer system, calculated at a bias voltage of 0.8 V. This value is chosen to account for the entire low energy edge contribution to the transmittance probability (see insets of Figure 2 and Section S4, Supporting Information). To study the edge‐to‐edge transport properties, we focus on large lateral displacements that constitute the final 3 nanometers before full removal of the upper flake from its lower counterpart. The calculated current fluctuations are in very good agreement with the experimental data. In particular, both the oscillation magnitudes (up to 10–20 µA) and their period (≈4.7 Å) are nicely reproduced, suggesting that the observed trend is inherent to the interface edges and is not strongly affected by the overall system dimensions (which are considerably higher in the experiment) or by the specific degree of edge overlap between the graphitic contacts. To verify that the observed current oscillations, manifesting the quantum mechanical interference of interlayer edge states, are robust against edge reconstruction effects, we repeated some of the transport calculations using geometrically relaxed graphitic bilayer interfaces. The results, presented in Section S6, Supporting Information, confirm that apart from some minor variations in their pattern, the period and magnitude of the current oscillations in edge reconstructed interfaces remain unchanged and are dominated by edge state contributions. In addition, we note that the edges of the various layers within the graphitic stack are most probably passivated by a variety of chemical terminations, in particular by different edge oxidation schemes as a result of the oxygen based etching process. This issue has been previously studied, demonstrating that zigzag edge states survive various edge‐oxidation schemes, and their effect may even be enhanced by edge polarization.^[^ ^23^ ^]^ Therefore, we do not expect that edge chemistry (and especially edge oxidation) will influence the qualitative nature of our general conclusions regarding interlayer edge transport and current oscillations.

The geometrical contribution to the current variations can be evaluated by calculating the registry index (RI) of the interface^[^ ^6^, ^38^ ^]^—a metric that quantifies the degree of interlayer lattice mismatch.^[^ ^38^, ^39^, ^40^, ^41^, ^42^, ^43^ ^]^ The RI variations (baseline removed, see Section S5, Supporting Information for further details) with lateral shifts of the same twisted bilayer system are plotted in Figure 3c. It is evident that interlayer lattice registry variations alone are insufficient to rationalize the calculated current profile of Figure 3a as they present a substantially shorter oscillation period ( < 2 Å) compared to the calculated and measured current profiles. In contrast, the variations in the summed atomic‐centered transmittance‐weighted Gaussian overlaps, presented in Figure 3b, show good correspondence with the current profile. Specifically, at lateral shifts of 89.7 and 95.45 Å, for which the calculated current exhibits local minima, the integrated Gaussian overlaps are considerably smaller than for the higher current interlayer configurations shifted by 92.12 and 96.97 Å.

**Figure 4:**
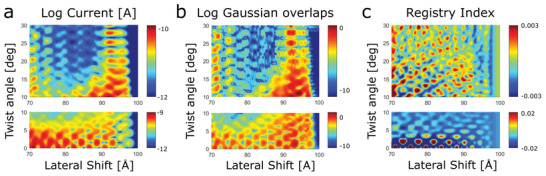
Computational investigation of interlayer current and registry variations as a function of inerlayer twist angle and lateral shifts in graphitic interfaces at the edge contact regime. a) Vertical current as a function of lateral shift and twist angle (measured with respect to Bernal stacking), calculated at a bias voltage of 0.8 V for a 10 nm diameter circular bilayer graphene system with a fixed interlayer distance of 3.35 Å. b) Lateral position and twist angle dependence of the integrated overlap between the two‐dimensional Gaussians representing the atomic‐centered transmittance‐weighted MO coefficients of the upper and lower flakes within the Fermi transport window. c) Registry index variations with lateral shift and twist angle. The moderately varying RI background was subtracted in order to clearly present the RI fluctuations (see Section S5, Supporting Information for further details). A lateral distance of 0 Å indicates that the flakes are completely overlapping, whereas at a lateral distance of 100 Å the upper flake is fully removed from its lower counterpart. Separate color bar scales are used in the low (0°–10°) and high (10°–30°) twist angle regimes to emphasize the variation patterns of the plotted quantities.

To study the effect of the twist angle on the electronic interference patterns and their manifestation in the edge‐state transport, we repeated the calculations for other twist angles in the full range of 0°–30°. In **Figure** **4** we present two‐dimensional plots of the dependence of the calculated (a) vertical current, (b) Gaussian overlaps, and (c) RI on the lateral displacement and interlayer twist angle. It is evident that the conclusions made above for the 10° twisted interface hold true for all other twist angles: geometrical registry arguments (Figure 4c) are unable to capture the fine details of the electronic transport variations (Figure 4a) indicating that quantum mechanical interlayer electronic coupling effects (Figure 4b) play a central role in edge‐transport scenarios. Specifically, we find that the magnitude of the interference‐induced edge‐state current fluctuations reduces with increasing twist angle, which is consistent with the corresponding reduction in the overall lattice registry between the layers. Nevertheless, the correlation between the calculated current fluctuations and the registry index is quite low for all angular configurations. Hence, the variations of the transmittance‐weighted wave function overlaps with the stacking mode must be accounted for to rationalize the calculated displacement induced current fluctuations.

## 3 Conclusion

Our findings support recent understanding that the design of graphene‐based mesoscopic electronic devices requires special attention to the appearance of edge states that may alter and, under certain circumstances, even govern their performance.^[^ ^22^ ^]^ Importantly, we find that geometrical lattice registry considerations are insufficient to explain the intricate dependence of interlayer edge transport on the stacking mode and twist angle of the interface and that substantial quantum mechanical interference effects, occurring over an edge overlap range smaller than 5 nm, may dictate the overall edge transport characteristics of the system. One, therefore, must carefully account for such effects when laying out nanoelectronic architectures based on confined graphene interfaces.

## 4 Experimental Section

### Fabrication of Mesoscale Graphitic Contacts

The experiments were carried out on cylindrical shaped mesa structures with a diameter of ≈ 200 nm and a typical total height of ≈ 100 nm, similar to previous studies.^[^ ^10^, ^31^ ^]^ The mesas were fabricated from a high quality highly oriented pyrolytic graphite (HOPG) substrate by means of electron‐beam lithography and metal lift‐off deposition for the fabrication of Pd‐Au masks followed by O_2_ reactive ion etching (RIE). The metal contact comprises of Pd‐Au metal layers of 10–40 nm, respectively. The RIE was set to etch down ≈ 50 nm of the unprotected graphite, resulting in a pillar structure.

### Mechanical Manipulation and Charge Transport Measurements

The lateral sliding of the top mesa contact was perform using a tip velocity of 50 nm s^−1^, where the applied voltage to the Pt‐Ir coated AFM tip was set to 2.5 V. The lateral shear force was measured during the sliding process in order to verify that sliding is performed under superlubric conditions (see Section S1, Supporting Information).^[^ ^10^ ^]^ The applied normal force during the slide was set to zero to avoid out‐of‐plane mesa deformation that can potentially affect the interlayer conductance, in particular toward the end of the slide. The interface conductivity was extracted based on a numerical fit to an equivalent electrical circuit. This circuit includes the constant serial resistances of the bulk graphite mesa structures, tip‐sample contact, and spreading resistance of the graphite substrate, whereas the sliding interface is represented by two lateral shift dependent parallel resistors corresponding to the charge flow through the area and circumference of the sliding contact (see Section S2, Supporting Information).^[^ ^22^ ^]^

### Electronic Transport and Registry Index Calculations

To evaluate the interlayer electronic transport behavior of the graphitic interface, circular bilayer graphene junction models with a diameter of 10 nm and various twist angles and lateral shifts were constructed. It is noted that no chemical edge passivation was employed as the TB model does not account for σ orbitals, which are much lower in energy than π orbitals. The electronic structure of the various junctions was modeled using the tight‐binding approximation with a Hamiltonian that includes an exponentially decaying interlayer hopping integral between carbon atoms residing on adjacent layers (see Section S3, Supporting Information).^[^ ^6^, ^36^ ^]^ The energy‐dependent electronic transmittance probability through the system was evaluated using non‐equilibrium Green's function theory and the Landauer formalism was used to evaluate the interlayer transport through the bilayer system.^[^ ^37^ ^]^ Further details regarding the calculations are provided in Section S4, Supporting Information. Transmittance weighted molecular orbital maps were obtained by the following procedure: (i) calculating the molecular orbitals of each bilayer configuration via diagonalization of the corresponding Hamiltonian; (ii) for each eigenstate residing within the Fermi transport window (set by the external bias potential) its MO expansion coefficients were multiplied by the transmittance probability evaluated at the corresponding eigenvalue energy, thus effectively weighing each MO according to its contribution to the transport process; (iii) assigning each atomic site a two‐dimensional Gaussian of fixed height, whose standard deviation is set proportional to the sum of transmittance‐weighted coefficients, associated with the given atomic position, of all MOs residing within the bias window. Further details regarding the calculation of the transmittance weighted molecular orbital maps and the Gaussian overlaps are given in Section S3, Supporting Information.

To quantify the degree of geometrical interlayer lattice matching in the studied bilayer systems the registry index method was utilized.^[^ ^38^, ^39^, ^41^, ^42^, ^43^ ^]^ In this approach, each carbon atom was assigned a Gaussian with an amplitude of 1 and a standard deviation equal to 0.75 of the carbon–carbon bond length.^[^ ^43^ ^]^ At each interlayer position, the projected overlaps of Gaussians associated with atoms in the upper layer and those of atoms in the lower layer were calculated and summed. In the present study, the summed overlap was normalized to that of the eclipsed Bernal stacking configuration. When plotting the RI at different interlayer stacking modes, the slowly varying background (based on a 3rd order polynomial fit) was subtracted in order to better observe the RI oscillations associated with edge overlap variations. Further details regarding the RI calculations are given in Section S5, Supporting Information.

## Conflict of Interest

The authors declare no conflict of interest.

## Supporting information

Supporting Information

Supplemental Movie 1

## Data Availability

The data that support the findings of this study are available from the corresponding author upon reasonable request.
