# Supplementary material for: Edge State Quantum Interference in Twisted Graphitic Interfaces
Source: Adv Sci (Weinh). 2022 Mar 13;9(14):2102261. doi: 10.1002/advs.202102261 (PMC9108635; doi:10.1002/advs.202102261)
Supplement: Supplementary file 1 — Supporting Information [file ADVS-9-2102261-s001.pdf]

ADVANCED SCIENCE
Open Access

Supporting Information

for *Adv. Sci.*, DOI 10.1002/advs.202102261

Edge State Quantum Interference in Twisted Graphitic Interfaces

Annabelle Oz, Debopriya Dutta, Abraham Nitzan, Oded Hod and Elad Koren**

Supporting Information

Edge State Quantum Interference in Twisted Graphitic Interfaces

Annabelle Oz[1ǂ], Debopriya Dutta,[2ǂ] Abraham Nitzan,[1,3] Oded Hod,[1*] and Elad Koren[2*]

[1]*Department of Physical Chemistry, School of Chemistry, The Raymond and Beverly Sackler Faculty of Exact Sciences and The Sackler Center for Computational Molecular and Materials Science, Tel Aviv University, Tel Aviv, IL 6997801.*

[2]*Faculty of Materials Science and Engineering and the Russell Berrie Nanotechnology Institute, Technion – Israel Institute of Technology, 3200003 Haifa, Israel.*

[3]*Department of Chemistry, University of Pennsylvania, Philadelphia, PA, USA 19103*

The supporting information contains the following items:

1. Lateral force measurements
2. Interface conductivity
3. Calculation of Gaussian overlaps
4. Electronic transport calculations
5. Registry Index calculations
6. Robustness against edge reconstruction

ǂ Equal authors contribution

*Corresponding authors: *eladk@technion.ac.il* ; *odedhod@tau.ac.il*

1. Lateral force measurements

The lateral shear force was measured during the sliding process in order to verify that sliding is performed under superlubric conditions, thus ensuring the existence of an angular mismatch at the bilayer graphene interface[1] (Fig. S1a). The shear force was evaluated using the relation $F = 2\sigma r$, applicable for small shear distances,[1] where r is the mesa radius and $\sigma = 0.227\, J{\cdot}m^{-2}$ is the adhesion energy of graphite.[1] The measured lateral force oscillations were smaller than $10\, nN$ indicating that sliding is indeed performed under superlubric conditions and that there is a rotational mismatch of $5° - 15°$ between the bottom and top graphene layers.[1,2]

2. Interface conductivity

A numerical fitting procedure was employed to obtain the current vs. sliding distance profile, $I(x)$, and to extract the corresponding resistance of the sheared interface, R_{int}, and its Edge (*E*) and Area (*A*) contributions (Fig S1b). $I(x)$ was calculated based on the equivalent electrical circuit depicted in the inset of Fig. S1b, i.e. $I(x) = \frac{V_{applied}}{[2\times R_{Gr}+R_{sys}+R_{int}]}$, where $V_{applied} = 2.5$ V is the applied voltage, R_{Gr} is the average resistance of the upper and lower graphene stacks, R_{sys} is the resistance of the electronic system consisting the electrical cables and AFM circuitry, and $R_{int} = \left[\left(R_{int}^{Area}\right)^{-1} + \left(R_{int}^{Edge}\right)^{-1}\right]^{-1}$ is the interface resistance composed of the surface $\left(R_{int}^{Area}\right)$ and edge $\left(R_{int}^{Edge}\right)$ components, assumed to contribute as parallel conductors.

The area and edge interfacial resistances are related to the lateral sliding distance, *x*, via $R_{int}^{Area} = \frac{\rho^{Area}}{S^{Area}(x)}$ and $R_{int}^{Edge} = \frac{\rho^{Edge}}{L^{Edge}(x)}$, where contact surface area and edge length are given by $S^{Area}(x) = 2\left(r^2 \cdot cos^{-1}\left(\frac{x/2}{r}\right) - \frac{x}{2}\sqrt{r^2 - \left(\frac{x}{2}\right)^2}\right)$ and $L^{Edge}(x) = 4\left(r \cdot cos^{-1}\left(\frac{x/2}{r}\right)\right)$, respectively, where r is the radius of the circular mesa and the corresponding resistivities ρ_{Area} and ρ_{Edge} serve as fitting parameters.

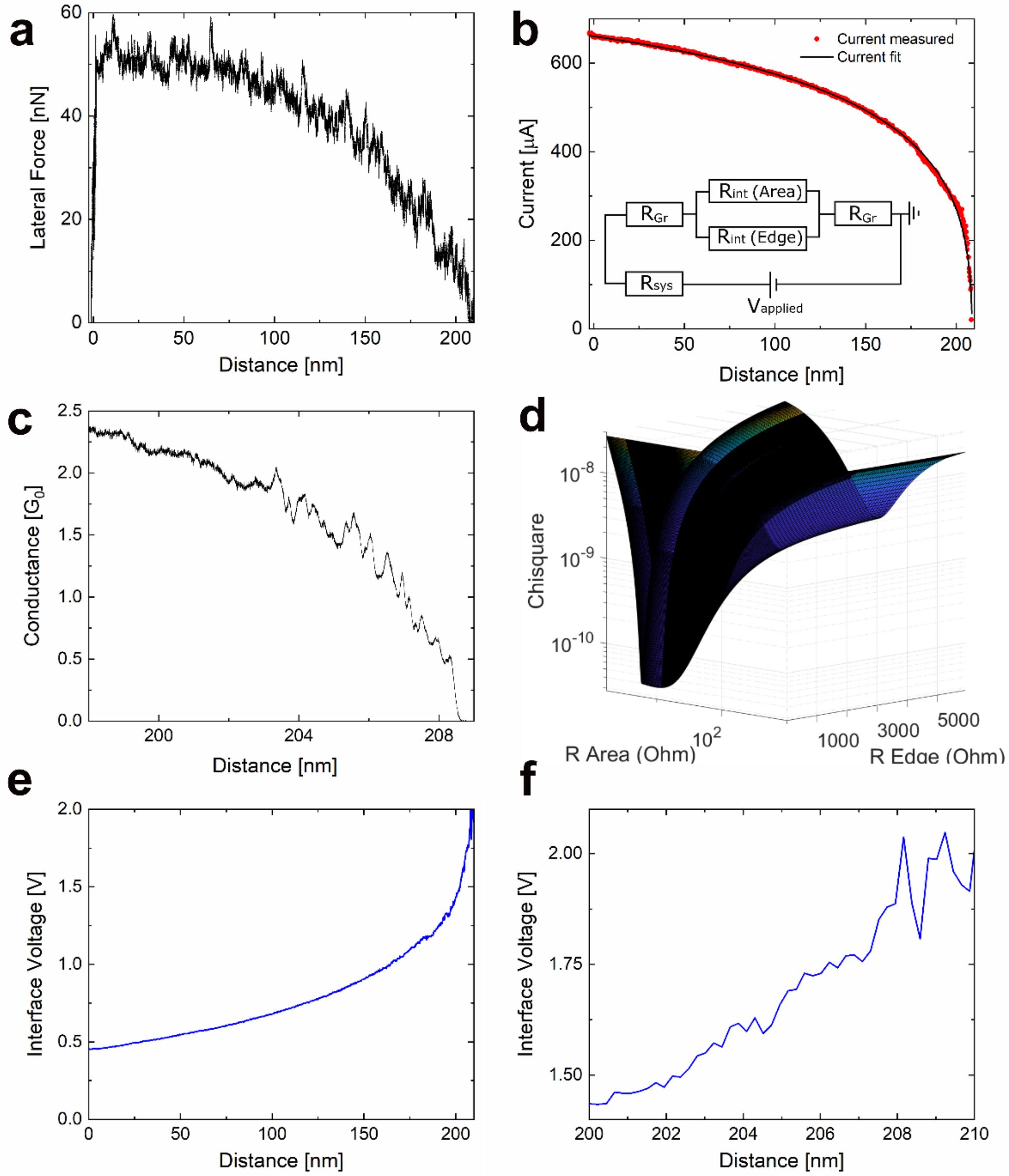

Figure S1: **(a)** Measured lateral force as a function of lateral displacement. Inset shows a schematic illustration of the sheared mesa from top view marking the overlap area (A) and edge (E) parts. **(b)** Measured current (red line) and corresponding numerical fit (black) based on the equivalent electrical circuit shown in the inset, which includes constant serial resistors (R_{sys}, 2x R_{Gr}) and two variable parallel resistors $\left(R_{int}^{Area},\ R_{int}^{Edge}\right)$ comprising the sheared interface. **(c)** Interface conductance (given in units of the conductance quantum G_0) as a function of lateral interfacial shift. **(d)** χ^2 diagram of the numerical fit to the experimental data presented in panel (b) as a function of area and edge resistance values. The combination $R_{Area} = 3620\ \Omega$ and $R_{Edge} = 1175\ \Omega$ (corresponding to the interface's area and edge resistances at the fully eclipsed configuration, respectively) that produces the lowest χ^2 value is used to extract ρ^{Area} and ρ^{Edge} that yield the black line fit appearing in panel (b). **(e)** Voltage drop across the twisted bilayer interface. **(f)** zoom-in on the last 10 nm region of (e).

The values of R_{Gr} and R_{sys} are considered to be constant throughout the sliding. Figure 1Sc presents the interface conductance as a function of lateral distance in units of the conductance quantum G_0. The quality of the numerical fit is assessed based on a χ^2 test, where the average sum of the differences between fitted and measured current, $\chi^2 = \frac{1}{n}\sum_{i=1}^{n}\left(I_i^{fit} - I_i^{meas}\right)^2$, is plotted for a wide range of relevant edge and area resistances and the values minimizing χ^2 are chosen as the optimal fit (Fig 1Sd). Here, I_i^{fit} and I_i^{meas} are the ith fitted and measured current datapoint, respectively and n is the total number of data points. The potential drop across the sheared interface (Fig. S1e,f) can be evaluated as the ratio of the total current (see Fig. S1b) and the interface conductance (Fig. S1c).

3. Calculation of Gaussian overlaps

As stated in the main text, in order to rationalize the measured current oscillations with lateral interfacial shifts in terms of variations of the bilayer's wave function, we constructed 10° twisted (with respect to the Bernal stacking) circular bilayer graphene models and described their electronic properties using a tight-binding Hamiltonian. The Hamiltonian includes one P_z orbital per carbon atom, describing the π band of each flake and an exponentially decaying inter-layer hopping integral describing the inter-flake electronic coupling, as follows:[3,4]

$$H_{ij} = \begin{cases} 0.5eV_b & i = j \in Top\,flake \\ -0.5eV_b & i = j \in Bottom\,flake \\ t_{cc} & i,j \neq i \text{ nearest neighbors on the same graphene layer} \\ t_p \cdot \exp\left\{-\frac{|d|-T_{vz}}{L_z}\right\} \cdot \exp\left\{-\left(\frac{r_{xy}}{L_{xy}}\right)^{\alpha}\right\} & i,j \neq i \text{ reside on adjacent graphene layers} \\ 0 & \text{Otherwise} \end{cases}, \quad \text{(S1)}$$

where $V_b = 0.8\,V$ is the externally applied bias voltage; e – is the electron charge; $t_{cc} = 3.16$ eV is the graphene intralayer nearest neighbors hopping integral; $t_p = 0.39$ eV is the interlayer hopping integral, $|d|$ and r_{xy} are the absolute and lateral distances between atoms i and j residing on adjacent graphene layers (given in Å), respectively; $T_{vz} = 3.35$ Å is the equilibrium interlayer distance in graphene; $L_{xy} = 1.7$ Å and $L_z = 0.6$ Å are the lateral and vertical interlayer interaction decay lengths, respectively; and $\alpha = 1.65$. We note that in this treatment Coulomb addition energies, observed in narrow (< 50 nm) graphitic systems [5–7] are not account for. This approximation is well justified for the large graphitic contacts considered in our experiments (> 200 nm) and in light of previous convincing experimental evidence observing pronounced zigzag edge state in the vicinity (< 100 mV) of the Fermi energy [8,9].

For each stacking configuration we first calculated the interlayer electronic transmittance probability, $T(E)$, using the non-equilibrium Green's function formalism (see SI section 4 below)[3,4] based on the above tight-binding Hamiltonian. Next, we calculated the molecular orbitals (MOs) of the full bilayer system by diagonalizing the corresponding Hamiltonian. The obtained MOs, $\{\psi_l\}$, are given by $\psi_l = \sum_i c_i^l P_Z^i$, where c_i^l is the contribution of atomic orbital P_Z^i to MO ψ_l ($\left|c_i^l\right|^2$, represents the probability of an electron occupying MO ψ_l to be found on atomic site i, assuming orthogonal atomic orbitals), and the corresponding eigen-energies are denoted by $\{\varepsilon_l\}$. Next, for each eigenstate, ψ_k, residing within the Fermi transport window ($E_F - 0.5eV_b < \varepsilon_k < E_F + 0.5eV_b$, where E_F is the Fermi energy of the entire bilayer flake and we assume, for simplicity, zero electronic temperature) we multiplied its absolute squared MO

expansion coefficients $\left|c_i^k\right|^2$ by the transmittance probability evaluated at the corresponding eigenvalue energy, $T(\varepsilon_k)$. This effectively weighs each MO according to its contribution to the transport process.

Since the tight-binding Hamiltonian does not involve explicit atomic orbitals, in order to visualize the interlayer overlap between the transmittance-weighted MOs, we assigned each atomic site a two-dimensional Gaussian of fixed height, centered around its position $\boldsymbol{R}_i = (x_i, y_i)$:

$$G_i(\boldsymbol{r} - \boldsymbol{R}_i) = e^{-\frac{|r-\boldsymbol{R}_i|^2}{2\sigma_i^2}} = e^{-\frac{(x-x_i)^2+(y-y_i)^2}{2\sigma_i^2}}, \quad \text{(S2)}$$

whose standard deviation is set proportional to the sum of transmittance-weighted coefficients of all MOs within the bias window associated with this atom:

$$\sigma_i = \frac{FWHM}{2\sqrt{2ln2}}\left[\sum_{k\,\in\,\text{Fermi Window}}\left|c_i^k\right|^2 T(\varepsilon_k)\right]^{\frac{1}{2}}, \quad \text{(S3)}$$

where we choose $FWHM = L_{CC}$ (where $L_{CC} = 1.42$ Å is the C-C bond length) such that the typical Gaussians extent matches the lattice spacing and a relatively smooth Gaussian overlap curve is obtained (see Fig. S2). The square root appearing in Eq. (S3) sets the two-dimensional Gaussian typical area (rather than its standard deviation), used later in the overlap calculations, to be proportional to the transmittance-weighted electronic contribution on each site.

Finally, the overlap between the Gaussians associated with atomic position i in one layer and atomic position j in the other layer is given by:

$$S_{ij} = \int_{-\infty}^{\infty} e^{-\frac{|r-\boldsymbol{R}_i|^2}{2\sigma_i^2}} e^{-\frac{\left|r-\boldsymbol{R}_j\right|^2}{2\sigma_j^2}} d\boldsymbol{r} = \frac{2\pi\sigma_i^2\sigma_j^2}{\sigma_i^2+\sigma_j^2} e^{\frac{-\left|\boldsymbol{R}_i-\boldsymbol{R}_j\right|^2}{2\left(\sigma_i^2+\sigma_j^2\right)}}. \quad \text{(S4)}$$

An example of the sum of Gaussian overlaps as a function of lateral inter-flake distance for three different FWHM values is presented in Fig. S2.

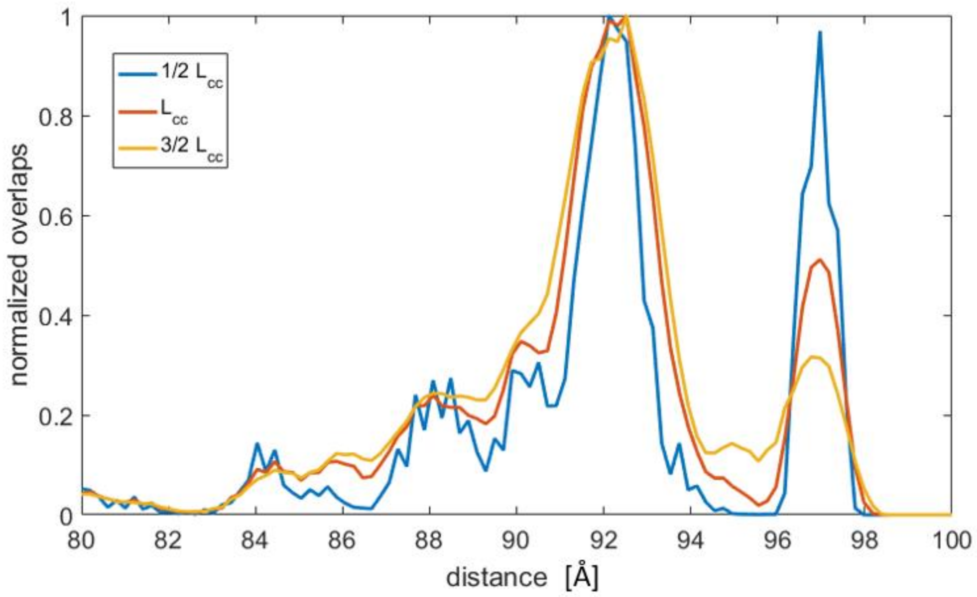

Fig. S2: Sum of overlaps between two-dimensional Gaussians representing transmittance-weighted MO contributions on the different atomic sites, normalized to the maximal value in the presented range, as a function of the lateral distance between two 10° twisted circular flakes of diameter $d = 10$ nm, using $FWHM = \frac{1}{2}L_{cc} = 0.71$ Å, $FWHM = L_{cc} = 1.42$ Å, $FWHM = \frac{3}{2}L_{cc} = 2.13$ Å. The Fermi window over which MOs are summed is set to $\pm 0.4\ eV$ and the transmittance probability is evaluated using broadening factors of $\Gamma_T = \Gamma_B = 0.05$ eV (see SI section 4 below).

4. Electronic transport calculations

The calculation of the interlayer transport of the graphitic junction is described in details in the supporting information of Refs. 3,4, and given here as well for the sake of completeness. The model junction comprises of two finite circular graphene flakes. The current passing from the upper flake to the lower flake is evaluated via the Landauer scattering formalism[10] that relates the current, I, to the transmittance probability of an electron through the system, $T(E)$ via:

$$I = \frac{2e}{h}\int [f_T(E) - f_B(E)]T(E)\mathrm{d}E. \qquad \text{(S5)}$$

In Eq. (S5), e is the electron charge, h is Plank's constant, and

$$f_{T/B}(E) \equiv f_{T/B}\left(E;\mu_{T/B},\beta_{T/B}\right) = \left[1 + e^{\beta_{T/B}\left(E-\mu_{T/B}\right)}\right]^{-1} \qquad \text{(S6)}$$

are the equilibrium electronic Fermi-Dirac distributions of the top/bottom leads. In Eq. (S6), $\beta_{T/B} = (k_B T_{T/B})^{-1}$ are the inverse electronic thermal energies of the top (T) and bottom (B) leads and $T_{T/B}$ are the corresponding electronic temperatures. The chemical potentials of the leads are taken to be $\mu_{T/B} = E_F \pm 0.5eV_b$, such that they evenly split the bias voltage, V_b, around the ground state Fermi energy of the entire finite model system, E_F. The factor of two appearing in Eq. (S5) accounts for spin degeneracy. In the present treatment, we neglect the effect of the electric field drop across the junction, due to the externally applied bias voltage, on the transmittance probability. However, we shift the onsite energies of the top and bottom lead atoms up or down, according to the corresponding lead chemical potential (see Eq. S1 in SI section 3 above). The transmittance probability $T(E)$ appearing in Eq. (S5), is calculated using the non-equilibrium Green's function formalism, which for elastic electronic transport gives:

$$T(E) = \mathrm{Tr}[\boldsymbol{G}_d^r(E)\boldsymbol{\Gamma}_B(E)\boldsymbol{G}_d^a(E)\boldsymbol{\Gamma}_T(E)]. \tag{S7}$$

Here, $\boldsymbol{\Gamma}_{T/B}(E)$ are the top and bottom broadening matrices described below, and $\boldsymbol{G}_d^{r/a}(E)$ are the retarded (r) and advanced (a) Green's function matrix representations of the junction given by:

$$\boldsymbol{G}_d^r(E) = [E\boldsymbol{I} - \boldsymbol{h}_d - \boldsymbol{\Sigma}_B^r(E) - \boldsymbol{\Sigma}_T^r(E)]^{-1}, \tag{S8}$$

and

$$\boldsymbol{G}_d^a(E) = [\boldsymbol{G}_d^r(E)]^{\dagger}, \tag{S9}$$

where $\boldsymbol{I}$ is a unit matrix of dimensions of the system and $\boldsymbol{h}_d$ is the matrix representation of the device's Hamiltonian given in the following block form:

$$\boldsymbol{h}_d = \begin{pmatrix} \boldsymbol{h}_T & \boldsymbol{v}_{TB} \\ \boldsymbol{v}_{BT} & \boldsymbol{h}_B \end{pmatrix}. \tag{S10}$$

In Eq. (S10), $\boldsymbol{h}_{T/B}$ are the Hamiltonian blocks of the top and bottom flakes given in the tight-binding atomic basis representation and $\boldsymbol{v}_{BT} = \boldsymbol{v}_{TB}^{\dagger}$ are their mutual coupling matrices. Note that in the tight-binding representation the atomic orbitals are assumed to be orthogonal and the overlap matrix is the unit matrix of the full system dimensions.

The leads self-energies are approximated under the wide band approximation as energy independent diagonal matrices providing the same lifetime, γ^{-1}, for each atomic site of the corresponding flake:

$$\boldsymbol{\Sigma}_T^r = i\hbar\gamma \begin{pmatrix} \boldsymbol{I}_T & \boldsymbol{0} \\ \boldsymbol{0} & \boldsymbol{0} \end{pmatrix}, \boldsymbol{\Sigma}_B^r = i\hbar\gamma \begin{pmatrix} \boldsymbol{0} & \boldsymbol{0} \\ \boldsymbol{0} & \boldsymbol{I}_B \end{pmatrix}, \tag{S11}$$

and $\boldsymbol{\Sigma}_{T/B}^a = (\boldsymbol{\Sigma}_{T/B}^r)^{\dagger}$. The broadening matrices in Eq. (S7) are given in terms of the self-energies as:

$$\begin{cases} \boldsymbol{\Gamma}_T & = i[\boldsymbol{\Sigma}_T^r - \boldsymbol{\Sigma}_T^a] = i\left[i\hbar\gamma\begin{pmatrix}\boldsymbol{I}_T & \boldsymbol{0}\\ \boldsymbol{0} & \boldsymbol{0}\end{pmatrix} - (-i\hbar\gamma)\begin{pmatrix}\boldsymbol{I}_T & \boldsymbol{0}\\ \boldsymbol{0} & \boldsymbol{0}\end{pmatrix}\right] = -2\hbar\gamma\begin{pmatrix}\boldsymbol{I}_T & \boldsymbol{0}\\ \boldsymbol{0} & \boldsymbol{0}\end{pmatrix} \\ \boldsymbol{\Gamma}_B & = i[\boldsymbol{\Sigma}_B^r - \boldsymbol{\Sigma}_B^a] = i\left[i\hbar\gamma\begin{pmatrix}\boldsymbol{0} & \boldsymbol{0}\\ \boldsymbol{0} & \boldsymbol{I}_B\end{pmatrix} - (-i\hbar\gamma)\begin{pmatrix}\boldsymbol{0} & \boldsymbol{0}\\ \boldsymbol{0} & \boldsymbol{I}_B\end{pmatrix}\right] = -2\hbar\gamma\begin{pmatrix}\boldsymbol{0} & \boldsymbol{0}\\ \boldsymbol{0} & \boldsymbol{I}_B\end{pmatrix} \end{cases}. \tag{S12}$$

The broadening factor, $\hbar\gamma = 0.05$ eV, is chosen to be sufficiently large to obtain a smooth density of states of the top and bottom flakes to mimic their periodic counterparts. The results are tested to be insensitive to this choice (see supporting information section 5 of Ref. [2]).

To reduce the computational time, we transform Eq. (S7) to the diagonal basis of the dressed Hamiltonian:

$$\boldsymbol{H}_d^{\mathrm{r}} = \boldsymbol{h}_d + \boldsymbol{\Sigma}_B^r + \boldsymbol{\Sigma}_T^r, \tag{S13}$$

such that the transformation matrix $\boldsymbol{U}$ transforms the complex symmetric matrix $\boldsymbol{H}_d^{\mathrm{r}}$ to its diagonal representation $\widetilde{\boldsymbol{H}}_d^r$:

$$\widetilde{\boldsymbol{H}}_d^{\mathrm{r}} = \boldsymbol{U}^{-1}\boldsymbol{H}_d^r\boldsymbol{U}. \tag{S14}$$

By inserting $\boldsymbol{U}\boldsymbol{U}^{-1} = \boldsymbol{I}$ or its conjugate transpose between each pair of matrices in Eq. (S7) and using the cyclic property of the trace operation we obtain:

$$T(E) = \mathrm{Tr}[\boldsymbol{G}_d^r(\boldsymbol{U}\boldsymbol{U}^{-1})\boldsymbol{\Gamma}_B(\boldsymbol{U}\boldsymbol{U}^{-1})^\dagger\boldsymbol{G}_d^a(\boldsymbol{U}\boldsymbol{U}^{-1})^\dagger\boldsymbol{\Gamma}_T(\boldsymbol{U}\boldsymbol{U}^{-1})] =$$

$$\mathrm{Tr}\left[\underbrace{\boldsymbol{U}^{-1}\boldsymbol{G}_d^r\boldsymbol{U}}_{\equiv\widetilde{\boldsymbol{G}}_d^r}\underbrace{\boldsymbol{U}^{-1}\boldsymbol{\Gamma}_B(\boldsymbol{U}^{-1})^\dagger}_{\equiv\widetilde{\boldsymbol{\Gamma}}_B}\underbrace{\boldsymbol{U}^\dagger\boldsymbol{G}_d^a(\boldsymbol{U}^{-1})^\dagger}_{\equiv\widetilde{\boldsymbol{G}}_d^a}\underbrace{\boldsymbol{U}^\dagger\boldsymbol{\Gamma}_T\boldsymbol{U}}_{\equiv\widetilde{\boldsymbol{\Gamma}}_T}\right] = \mathrm{Tr}\left[\widetilde{\boldsymbol{G}}_d^r\widetilde{\boldsymbol{\Gamma}}_B\widetilde{\boldsymbol{G}}_d^a\widetilde{\boldsymbol{\Gamma}}_T\right]. \tag{S15}$$

In the new basis, the retarded and advanced Green's functions matrices still obey the required relation $\widetilde{\boldsymbol{G}}_d^a = \widetilde{\boldsymbol{G}}_d^{r\,\dagger}$, and have diagonal representations:

$$\widetilde{\boldsymbol{G}}_d^r = \left[E\boldsymbol{I} - \widetilde{\boldsymbol{H}}_d^r\right]^{-1}. \tag{S16}$$

This allows the evaluation of $\widetilde{\boldsymbol{G}}_d^r(E)$ and $\widetilde{\boldsymbol{G}}_d^a(E)$ at any value of E while avoiding repeated matrix inversions, at the expense of a single complex symmetric matrix diagonalization and a single evaluation of $\widetilde{\boldsymbol{\Gamma}}_B$ and $\widetilde{\boldsymbol{\Gamma}}_T$.

We note that, in the main text, all $T(E)$ calculations presented were performed over a window of $\pm 0.4\ eV$ around the Fermi energy of the entire bilayer model system. As can be seen in Fig. S1e,f, the experimental bias voltage drop across the twisted bilayer interface does not exceed $\sim 2\ V$. Fig. S3 below, demonstrates that within the corresponding energy window of $\pm 1.0\ eV$ around the Fermi energy, the major contribution arrives from a narrower region below $\pm 0.4\ eV$. Therefore, to reduce the computational burden, we opted to use this smaller energy window.

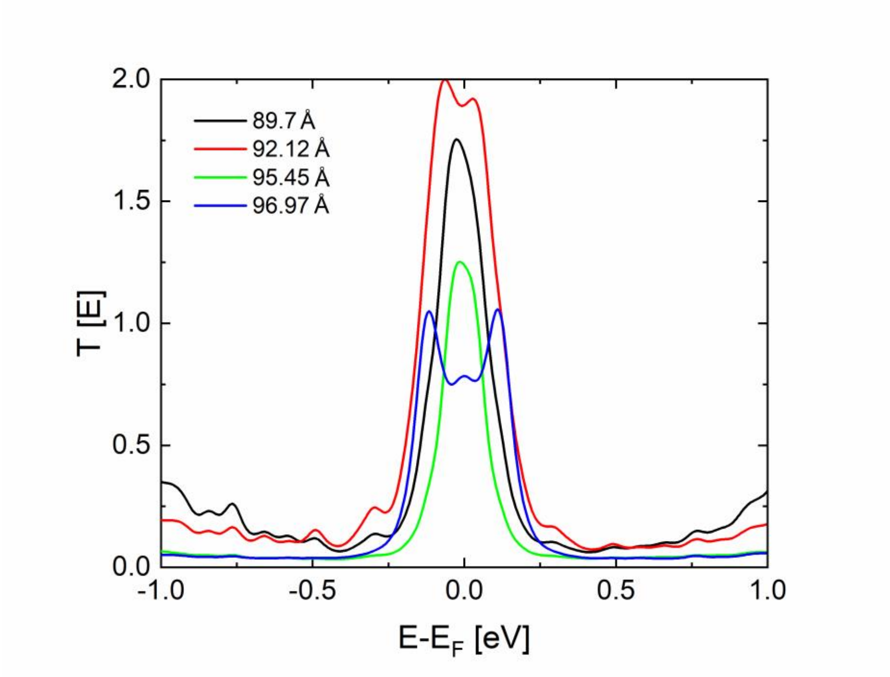

Fig. S3: Transmittance probability calculated for a 10 nm diameter 10° twisted circular bilayer graphene flake, over an extended energy window of $\pm 1\ eV$ around the Fermi energy of the entire bilayer mode, for four lateral positions corresponding to an interlayer shift of 89.7 Å (black line), 92.12 Å (red line), 95.45 Å (green line), and 96.97 Å (blue line).

5. Registry Index calculations

The registry index (RI) is a dimensionless quantity that quantifies the interlayer commensurability between two rigid interfaces, and has been used to model the interlayer sliding energy surfaces of a variety of hexagonal layered materials.[4,11–15]

In the original RI approach, developed for graphitic interfaces, each lattice atomic center is assigned a circle of radius r_i, which depends on the atomic identity.[14] To obtained smoother and more physical registry surfaces, the circles have been replaced in more recent calculations by two-dimensional atomic-centered Gaussian functions, whose standard deviations relate to the original circle radii as $\sigma_i = \gamma r_i$, where γ is chosen to reproduce reference sliding energy landscapes of graphitic interfaces and typically assumes a value of $\sigma_i = 0.75 L_{CC}$.[15] The projected Gaussian overlaps between atomic centers belonging to adjacent graphene surfaces are calculated according to:

$$S_{C_iC_j} = \frac{\pi\sigma_i^2\sigma_j^2}{\sqrt{\sigma_i^2+\sigma_j^2}} e^{-\frac{|r_i-r_j|^2}{2(\sigma_i^2+\sigma_j^2)}}, \tag{S17}$$

and summed to evaluate the degree of Pauli repulsive interactions, $S_{CC}^{tot} = \sum_{i \in Layer\ 1} \sum_{j \in Layer\ 2} S_{C_iC_j}$. This summation is then normalized to obtain the registry index:

$$RI = \frac{S_{CC}^{tot} - S_{CC}^{opt}}{S_{CC}^{worst} - S_{CC}^{opt}}, \tag{S18}$$

that is bound to the values of $RI = 0$ and $RI = 1$, obtained at the optimal (AB) and worst (AA) stacking modes, respectively.

While in its original implementation the registry index approach was developed to describe the interlayer sliding potential in rigid layered materials, it was later on extended to evaluate the qualitative behavior of the interlayer transport in graphitic junctions based on the degree of overlap between P_z orbitals associated with atoms residing on adjacent layers.[4] In the present study, we adopted this approach by setting $r_C = 0.1 \cdot r_{CC}$, where $r_{CC} = 1.42$ Å is the carbon-carbon bond length in graphene. Aiming to focus on RI variations, we remove the slowly varying RI baseline obtained during lateral sliding, based on a 3rd power polynomial fit to the calculations.

6. Robustness against edge reconstruction

To further confirm that the observed current oscillations, manifesting quantum mechanical interference of interlayer edge states, are robust against edge reconstruction, we have repeated some of our transport calculations using geometrically relaxed graphitic bilayer interfaces. To this end, we employed the FIRE energy minimization algorithm [16,17], as implemented in the LAMMPS[18] software, with a force tolerance for convergence set to 10^{-8} on individual hydrogen terminated 10 nm diameter circular graphitic flakes, whose intra-layer interactions were described by the second-generation reactive empirical bond order (REBO) potential [19], which gives an equilibrium C-C bond length of ~ 1.42039 Angstroms. The relaxed flakes were stacked at an interlayer distance of 3.35 Å to form a partially overlapping bilayer system, twisted by $10°$ from Bernal stacking, whose interlayer transport properties were calculated. We note that the states associated with the hydrogen terminations do not contribute to the tight-binding-based transport calculations, as their energy is well outside the Fermi transport region. The results, appearing in Fig. S4 below, demonstrate that apart from some minor variations in their pattern, the period and

magnitude of the current oscillations in edge reconstructed interfaces remain unchanged. Moreover, to validate that the current fluctuations are dominated by edge states transport, we plot in Fig. S4 also the individual edge and bulk current contributions (red and green curves, respectively), calculated using the procedure described in Ref. [2]. The results demonstrate that bulk transport plays a negligible role across the final few interfacial slide nanometers, supporting our conclusion that the observed current fluctuations are induced by edge state quantum interference as opposed to bulk transport.

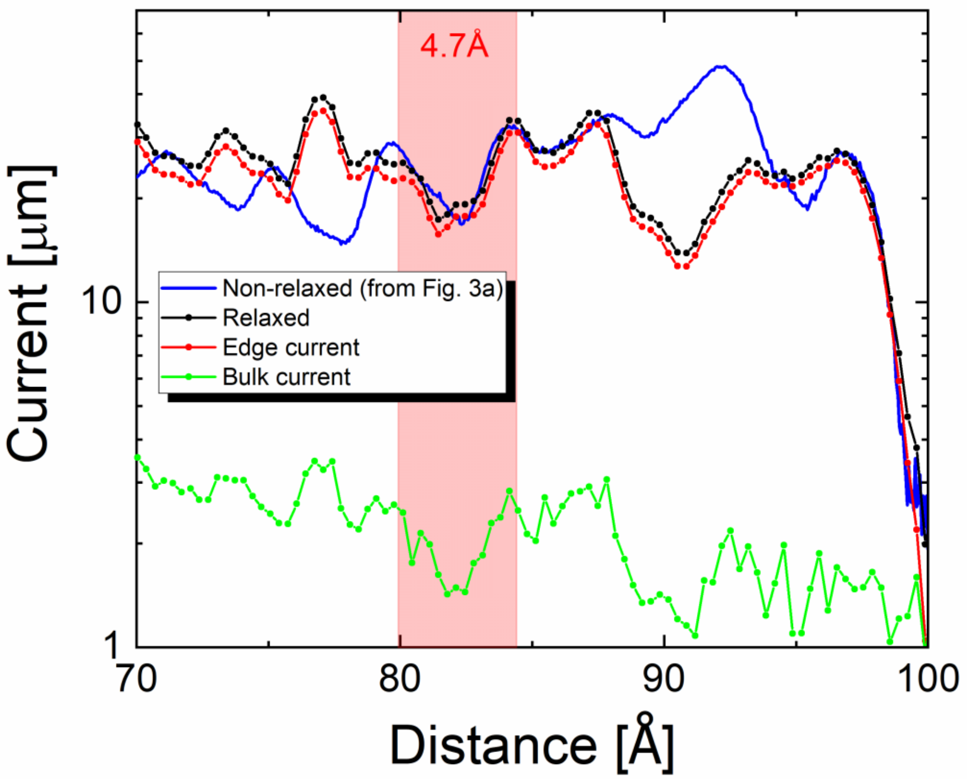

Fig. S4: Vertical current as a function of lateral shift, calculated with (black) and without (blue) edge reconstruction at a bias voltage of 0.8 V for a 10 nm diameter circular bilayer graphene system twisted by 10° from Bernal stacking with a fixed interlayer distance of 3.35 Å. The red and green curves represent the separate edge and bulk current contributions, respectively.

References

[1] E. Koren, E. Lortscher, C. Rawlings, A. W. Knoll, U. Duerig, *Science* **2015**, *348*, 679.

[2] D. Dutta, A. Oz, O. Hod, E. Koren, *Nature Communications* **2020**, *11*, 4746.

[3] K. M. M. Habib, S. S. Sylvia, S. Ge, M. Neupane, R. K. Lake, *Appl. Phys. Lett.* **2013**, *103*, 243114.

[4] E. Koren, I. Leven, E. Lörtscher, A. Knoll, O. Hod, U. Duerig, *Nature Nanotechnology* **2016**, *11*, 752.

[5] F. Sols, F. Guinea, A. H. C. Neto, *Phys. Rev. Lett.* **2007**, *99*, 166803.
[6] C. Stampfer, J. Güttinger, S. Hellmüller, F. Molitor, K. Ensslin, T. Ihn, *Phys. Rev. Lett.* **2009**, *102*, 056403.
[7] S. Dröscher, H. Knowles, Y. Meir, K. Ensslin, T. Ihn, *Phys. Rev. B* **2011**, *84*, 073405.
[8] Y. Kobayashi, K. Fukui, T. Enoki, K. Kusakabe, Y. Kaburagi, *Phys. Rev. B* **2005**, *71*, 193406.
[9] Y. Niimi, T. Matsui, H. Kambara, K. Tagami, M. Tsukada, H. Fukuyama, *Phys. Rev. B* **2006**, *73*, 085421.
[10] S. Datta, *Quantum Transport: Atom to Transistor*, Cambridge University Press, Cambridge, **2005**.
[11] N. Marom, J. Bernstein, J. Garel, A. Tkatchenko, E. Joselevich, L. Kronik, O. Hod, *Phys. Rev. Lett.* **2010**, *105*, 046801.
[12] O. Hod, *Israel Journal of Chemistry* **2010**, *50*, 506.
[13] A. Blumberg, U. Keshet, I. Zaltsman, O. Hod, *J. Phys. Chem. Lett.* **2012**, *3*, 1936.
[14] O. Hod, *ChemPhysChem* **2013**, *14*, 2376.
[15] I. Oz, I. Leven, Y. Itkin, A. Buchwalter, K. Akulov, O. Hod, *The Journal of Physical Chemistry C* **2016**, *120*, 4466.
[16] E. Bitzek, P. Koskinen, F. Gähler, M. Moseler, P. Gumbsch, *Physical Review Letters* **2006**, *97*, DOI 10.1103/PhysRevLett.97.170201.
[17] J. Guénolé, W. G. Nöhring, A. Vaid, F. Houllé, Z. Xie, A. Prakash, E. Bitzek, *Computational Materials Science* **2020**, *175*, 109584.
[18] A. P. Thompson, H. M. Aktulga, R. Berger, D. S. Bolintineanu, W. M. Brown, P. S. Crozier, P. J. in 't Veld, A. Kohlmeyer, S. G. Moore, T. D. Nguyen, R. Shan, M. J. Stevens, J. Tranchida, C. Trott, S. J. Plimpton, *Computer Physics Communications* **2022**, *271*, 108171.
[19] D. W. Brenner, O. A. Shenderova, J. A. Harrison, S. J. Stuart, B. Ni, S. B. Sinnott, *Journal of Physics: Condensed Matter* **2002**, *14*, 783.
